# Stochastic integer programming for multi-disciplinary outpatient clinic planning

**DOI:** 10.1007/s10729-017-9422-6

**Published:** 2017-11-09

**Authors:** A. G. Leeftink, I. M. H. Vliegen, E. W. Hans

**Affiliations:** 1 Center for Healthcare Operations Improvement and Research (CHOIR), University of Twente, P.O. Box 217, 7500 AE Enschede, the Netherlands; 2 UMC Utrecht Cancer Center, University Medical Center Utrecht, Utrecht, the Netherlands; 3 Department of Industrial Engineering & Innovation Sciences, Eindhoven University of Technology, Eindhoven, the Netherlands

**Keywords:** Multi-disciplinary planning, Stochastic processes, Sample average approximation, Appointment scheduling

## Abstract

Scheduling appointments in a multi-disciplinary clinic is complex, since coordination between disciplines is required. The design of a blueprint schedule for a multi-disciplinary clinic with open access requirements requires an integrated optimization approach, in which all appointment schedules are jointly optimized. As this currently is an open question in the literature, our research is the first to address this problem. This research is motivated by a Dutch hospital, which uses a multi-disciplinary cancer clinic to communicate the diagnosis and to explain the treatment plan to their patients. Furthermore, also regular patients are seen by the clinicians. All involved clinicians therefore require a blueprint schedule, in which multiple patient types can be scheduled. We design these blueprint schedules by optimizing the patient waiting time, clinician idle time, and clinician overtime. As scheduling decisions at multiple time intervals are involved, and patient routing is stochastic, we model this system as a stochastic integer program. The stochastic integer program is adapted for and solved with a sample average approximation approach. Numerical experiments evaluate the performance of the sample average approximation approach. We test the suitability of the approach for the hospital’s problem at hand, compare our results with the current hospital schedules, and present the associated savings. Using this approach, robust blueprint schedules can be found for a multi-disciplinary clinic of the Dutch hospital.

## Introduction

Multi-disciplinary teams are increasingly being introduced in various medical contexts, such as in outpatient clinics and operating rooms [24, 29], and in various medical disciplines, such as cancer care, rehabilitation, and neurology [15, 40, 47, 48]. However, the coordination and control of these teams is complex, as multiple clinicians from multiple departments are involved.

This research is motivated by University Medical Center Utrecht (UMCU), a large hospital in the Netherlands. During the redesign of one of their cancer outpatient clinics, a decision on the blueprint of the agendas of the involved nurse practitioners and clinicians has to be made. This is a complex decision, as multiple patient types are involved, and the overall performance of the cancer clinic depends on the interplay between all agendas. Therefore, the optimization of the blueprint schedules of this multi-disciplinary clinic requires an integrated optimization approach, in which all appointment schedules are jointly optimized.

The contribution of this paper is that we design optimized blueprint schedules for multi-disciplinary appointment planning at a tactical level of control, which incorporates uncertainties in patient routing. As this currently is an open question in the literature, our research is the first to address this problem. Also, we test the suitability of the approach for the hospital’s problem at hand, we compare our results with the current hospital schedules, and present the associated savings. Furthermore, although initiated from a cancer clinic application, many other multi-disciplinary applications can benefit from a solid approach towards multi-disciplinary clinic blueprint planning.

This paper is organized as follows: First, we introduce the problem in Section 2. Then, the relevant literature on open access multi-appointment planning is described in Section 3. Section 4 presents the mathematical problem description and solution method. Section 5 presents the proposed solution methodology, followed by the numerical experiments and a case study in Sections 6 and 7, respectively. Finally, Section 8 gives the conclusions, discussion and opportunities for further research.

## Problem description

**Fig. 1:**
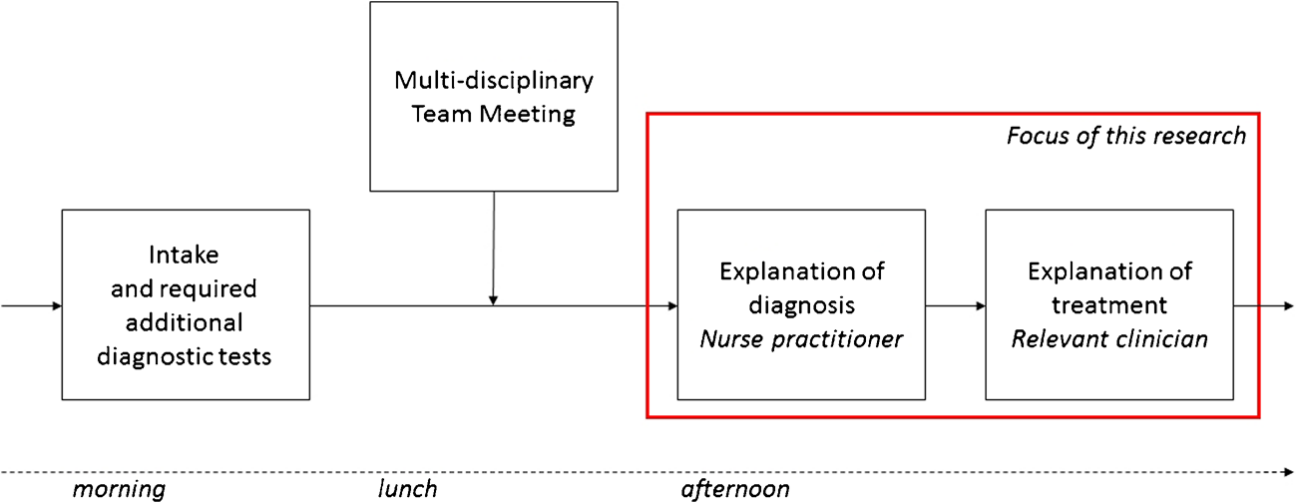
Diagnostic pathway of a cancer patient

Figure 1 shows the pathway of a cancer patient following the diagnostic trajectory in the hospital at hand at an arbitrary day in which a multi-disciplinary team meeting (MTM) takes place. In our collaborating hospital this is every Tuesday. These patients, with (a high probability of having) cancer, are often referred from other hospitals, and require multiple disciplines to be involved in their treatment. Therefore, this pathway starts with an intake, and if required some additional diagnostic tests, followed by an MTM. After the MTM, on that same day, the patient gets two consultations in the multi-disciplinary clinic. The first consultation is with a nurse practitioner (NP) (or another clinician, depending on the preference of the care system), where the patient receives the cancer diagnosis. Thereafter, the patient has a second consultation with a clinician who explains more about the proposed treatment. Each possible treatment is executed by a discipline, with corresponding clinicians who provide the treatment consultation. The treatment, and thus the type of clinician needed, is only known during the MTM. Therefore, there is uncertainty about the number of patients that require a consultation for each clinician type. In this paper we focus on these two consultations, which we will refer to as the `multi-disciplinary clinic’. The hospital aims to minimize waiting time between these two consultations, as patients receive a high-impact message from their care providers. Furthermore, the hospital wants their clinicians to be fully utilized. Therefore, the clinicians’ overtime and idle time need to be minimized as well.

The patients that follow this care pathway are referred to as `multi-disciplinary patients’. Thus, multi-disciplinary patients are patients that require an appointment with a NP, followed by a walk-in appointment with a clinician on a First Come First Serve (FCFS) basis. These multi-disciplinary patients are diagnosed for a specific tumor type. Similarly to practice, the schedule for the NPs is made directly following the MTM, thus the number of multi-disciplinary patients and the treatment modality for every patient is known at the time of scheduling their appointments with the NP. Therefore, the referral probabilities for a multi-disciplinary patient from the NP to the different clinician types are known. Furthermore, since multi-disciplinary patients are already in the hospital, the no-show rate of multi-disciplinary patients is close to 0 *%*. Therefore, we assume all multi-disciplinary patients will show up for their appointment with the NP.

Next to the multi-disciplinary patients, another patient type is admitted in the multi-disciplinary clinic, which we refer to as `regular patients’. Regular patients only require a pre-booked appointment with a specific clinician type, for example for a check-up appointment. These appointments are booked several weeks to months in advance. The regular patient demand is assumed to be sufficient to fill the maximum capacity of the clinic. We assume all regular patients to show up and to arrive on time for their appointment. Furthermore, they will be served on the time of their appointment, even if a multidisciplinary patient is waiting longer, as pre-booked appointments are prioritized. Since regular patients book their appointment in advance, the regular patient demand is known well before the multi-disciplinary patient demand. Therefore, schedulers need to know to which appointment slots in the clinicians’ agendas they can schedule regular patients, as selecting the wrong slots might lead to unnecessary idle, waiting, and overtime.

We aim to derive a planning method for scheduling multi-disciplinary patients in the agenda of the NPs, and a blueprint schedule for each of the clinicians which differentiates between slots for multi-disciplinary patients and regular patients. The NP schedules result in walk-in rates to the various clinician types. As various combinations of patients might result in various NP schedules, the clinicians’ blueprint schedules should be optimized together with all possible NP schedules.

For the agenda of the NPs we assume that the number of NPs, and thus the number of available appointment slots per time slot is known, that overbooking is not allowed, and that all slots are booked during every planning period. For the agenda of the clinicians, we assume that the number of clinicians per type are known, and that multi-disciplinary patients that walk-in into the waiting room of a clinician type, wait until the first available empty slot with any of the clinicians of that specific type. Regular patients are always served at the time of their appointment, and double-booked appointment slots are not allowed. We assume all patients are served, if needed in overtime, as one clinician per clinician type can work in overtime. Furthermore, as all clinicians agreed on the same service duration for all patients, no differentiation between service times of clinician types is required. The blueprint appointment schedule is designed as the number of appointments in the agenda of a clinician that can be booked for a regular patient for each time slot.

To evaluate the performance of the blueprint schedules, multiple objectives should be considered [26]. We consider the optimal schedule to be a schedule that minimizes a cost function, considering the expected multi-disciplinary patient waiting time between the two appointments, the clinician overtime, and the clinician idle time, similar to the cost function considered in [32]. The cost function is influenced by the number of regular patients to be scheduled in the clinicians’ schedules and their timing, as well as by the sequence in which multi-disciplinary patients are seen by the NPs.

**Fig. 2:**
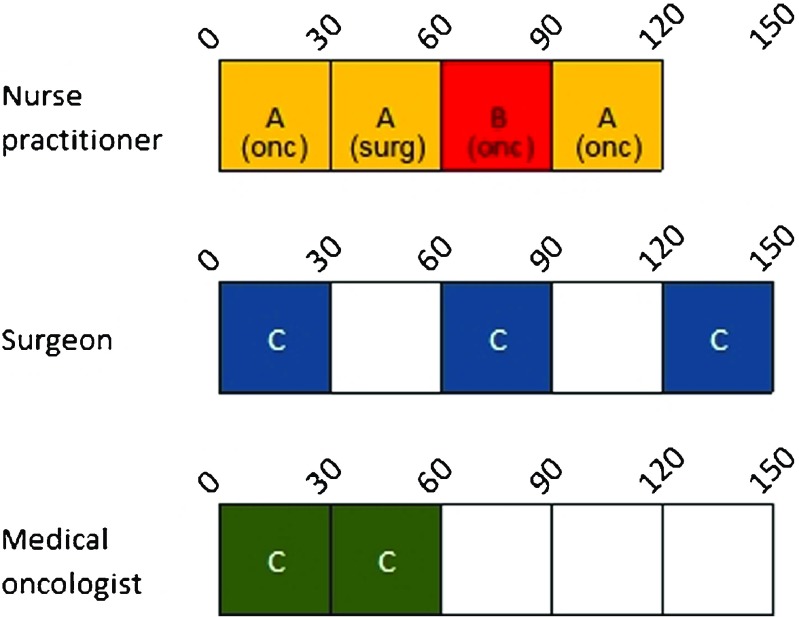
Example of a NP schedule and clinicians’ blueprint schedules of a small multi-disciplinary clinic with two clinicians. We consider consultations for multi-disciplinary patients with tumor type A (A), consultations for multi-disciplinary patients with tumor type B (B), and regular consultations (C). The empty slots in the clinicians’ schedules are available for multi-disciplinary patients on a FCFS basis

As an example, consider a very small multi-disciplinary clinic. Here, one NP has 4 time slots, and two clinicians of two clinician types (a surgeon and a medical oncologist) both have 5 time slots, as shown in Fig. 2. In this clinic, there are multi-disciplinary patients consulted with two tumor types. Multi-disciplinary patients with tumor A account for 1/4th of all multi-disciplinary patients seen, and have a probability of getting surgery of 20*%*, and a probability of getting chemotherapy of 80*%*. Multi-disciplinary patients with tumor B account for 3/4th of the multi-disciplinary patient population, and have a probability of getting surgery of 60*%*, and a probability of getting chemotherapy of 40*%*. The question is how many and in which time slots the clinicians can see regular patients, in order to minimize the expected waiting for multi-disciplinary patients, and to minimize the idle and overtime. Since regular patients want to get their appointment dates multiple weeks in advance, this schedule should be designed before the treatment modalities of the multi-disciplinary patients are known, as their treatment is decided during the MTM. However, at this point we do not know the number of arrivals of the two multidisciplinary patient types. Therefore, all possible optimal schedules of the NP should be taken into account as well, since these schedules determine the arrival rate to the clinicians. Following the MTM, after the treatment modalities of the multi-disciplinary patients are known, the optimal schedule for the NP can be determined and immediately executed, whereas the schedule for the clinicians is already fixed at that moment. An example of a possible schedule for the NP, and a possible blueprint schedule for the clinicians is shown in Fig. 2.

## Literature

Appointment scheduling has been well studied for many service industries, including health care [34]. Within the broad range of health care appointment scheduling literature, we focus on two particular areas. Section 3.1 focuses on multi-disciplinary scheduling, and Section 3.2 focuses on open access scheduling. In Section 3.3, we conclude by assessing the possibilities to combine multi-disciplinary and open access scheduling. For extensive overviews of appointment scheduling in health care, refer to [7, 16, 17].

### Multi-disciplinary scheduling

We define multi-disciplinary scheduling as the planning of multiple appointments per patient over multiple resource types. Although the implementation of multi-disciplinary systems has frequently been reported in the literature (e.g. [2, 13–15, 47]), the organization of these multi-disciplinary systems has been underrepresented in the literature [26].

This research focuses on tactical level appointment schedules for multi-disciplinary systems. The order of activities within these systems is fixed. Therefore, within the field of multi-disciplinary scheduling, we are interested in studies that include precedence relations, where jobs need to be scheduled in a predefined order on multiple resources. The system under review can be represented as a flow shop, which is known to be NP-hard for most configurations [12]. Multi-disciplinary open or mixed shop systems, such as [37], are out of scope.

Multiple authors consider the planning of flow-shop type multi-disciplinary systems, for example in oncology [23, 40] and primary care practices [30]. Liang et al. [23] analyze the impact of scheduling methods on the oncology clinic performance, where patients visit an oncologist and a nurse for chemotherapy treatment. Although various patient routings are considered, they consider this as given in their model. Saremi et al. [44] address the appointment scheduling of patients with various service sequences as well. They determine the appointment time of each patient in order to optimize a combination of waiting time and completion time. However, the number of patients per patient type, and thus the patient routing, are known in advance. Oh et al. [30] sequence patient appointments using a stochastic integer programming model with the sample average approximation method. They included the effects of uncertainty in service time, but fixed the patient routing requirements. Romero et al. [40] use simulation to evaluate different appointment scenarios in which appointment blocks are reserved for multi-disciplinary patients. This way, they prove the feasibility of a one-stop-shop for basal cell carcinoma. However, they do not optimize the amount of capacity that needs to be reserved for serving the multi-disciplinary patients.

Simulation is the most widely applied technique in the literature studying the organization of multi-disciplinary scheduling. Simulation is used to analyze the performance of multiple clinics under a variety of scenarios, including various appointment rules and appointment schedules [9, 18, 23, 25, 31, 40, 43, 44], which show significant improvements compared to the current practice in partnering health care centers.

Besides simulation, heuristics are applied. Both local search methods [42, 44] and other meta-heuristics [33] are applied to develop patient schedules, as well as approximate stochastic approaches [30] and simple planning rules [22, 41].

Since multi-disciplinary appointment scheduling involves multiple facilities that share patients, multiple performance indicators should be evaluated both for each facility at a local level as well as for the full system at a global level [26]. Not only the performance of the system, but also patient performance and clinician performance is taken into account in the literature.

Concluding, multi-disciplinary systems with precedence constraints are complex systems. Therefore, researchers focus on approximate solutions, such as simulation and heuristics, in order to optimize or evaluate the performance of these systems. To the authors’ knowledge, approaches to optimize or evaluate multi-disciplinary systems with stochastic patient routing are not available in the literature.

### Open access scheduling

Open access scheduling is also known as same day scheduling, advanced access scheduling, short-notice scheduling, and walk-in scheduling [17, 36]. It entails the planning of multiple patient classes with different planning horizons. Open access approaches are introduced by [28], and were quickly adopted to reduce the effect of no-shows and cancellations [38, 39], as an alternative to overbooking strategies [e.g. 11, 46]. Since this introduction, multiple researchers have researched the organization of open access appointment scheduling, both with a multi-day focus as well as an intra-day focus.

The multi-day focus concerns the percentage of appointment slots to reserve for open access patients [10, 35, 36, 45, 49], since this percentage influences amongst others the queue length and overtime [10]. Contrary to most available literature, Wiesche et al. [49] consider flexible capacity, to cope with varying patient arrival rates during the week in a primary care clinic. They use an integer programming approach to determine the optimal capacity, taking open access and regular patients into account, and evaluate the system performance by a stochastic simulation.

The intra-day focus concerns the sequence of fixed and open access appointments slots during the day [49], and the allocation of open access patients to appointment slots [4]. Since most authors evaluate multiple appointment sequences by a simulation study [e.g. 49, 19], only little work is performed on the optimization of these blueprint schedules. Peng et al. [32] Optimize the number and position of open access and regular appointments by developing a blueprint schedule that minimizes the patient waiting time and clinician idle and overtime. Due to the high problem complexity of real life cases, their Genetic Algorithm (GA) approach in combination with simulation requires high computational effort.

Few studies focus on the combination of multi-day and intra-day decisions. Kortbeek et al. [21] Optimize the blueprint schedule considering both walk-in and scheduled patients, while allowing walk-in patients to be deferred. They develop two queuing models and propose a heuristic approach to generate appointment schedules based on these models.

Most open access literature consider a single-provider service system [39] with fixed deterministic appointment intervals, where the capacity for each day is fixed and known [36], and where the demand and arrival rates are given [21, 35, 38]. The schedules of all providers involved are often assumed to be independent, both for providers of the same patient population, as well as for up- and downstream appointments [35, 36, 38]. Furthermore, a wide range of (combinations of) performance indicators is considered.

Concluding, open access scheduling focuses on determining the number of appointment slots and the sequencing of open access and fixed appointment slots. Where most literature assumes independent schedules, in our experience, schedules of clinicians influence each other, as the arrival rate at a walk-in clinic is determined by appointment schedules or service rates of upstream clinics. Open access scheduling with dependent schedules is an open challenge [36].

### Contribution

Despite the long tradition of appointment planning in the literature, there has not been any attention for developing blueprint scheduling for multi-disciplinary appointment planning with open access requirements, as multi-disciplinary clinics are an emerging area in health care.

The blueprint schedule design of a multi-disciplinary clinic with open access requirements requires an integrated optimization approach, in which all appointment schedules are jointly optimized. To the authors’ knowledge, the optimization of multiple clinics with open access requirements has not been considered before [as also argued by 6]. In addition, in all relevant literature, schedules of clinicians are assumed to be independent [see also 35, 36, 38], while in our experience with multiple hospitals, they depend on each other. Therefore, we analyze a multi-disciplinary clinic with open access requirements and dependencies between various clinicians tackling both open challenges.

Furthermore, we consider both multi-day and intra-day planning decisions. Since the sequencing of multi-disciplinary patients with the NPs influences the arrival rates at the clinician types, decisions on the available capacity for regular patients and on the slot sequencing of multi-disciplinary patients should preferably be made together.

Concluding, our contribution is threefold. First, we develop a model that includes dependent patient demand in open access models, an open challenge according to [36]. Second, multi-appointment planning in an open access context is considered in this model, an open challenge according to [6]. Third, practical applications are presented in a case study of a real life health care setting.

## Formal problem description and solution approach

To address the uncertainty in multi-disciplinary patient routing in the multi-disciplinary clinic, we adopt a stochastic programming approach. Stochastic programming has been applied in various health care settings. [27] developed a stochastic program to include uncertainty in surgery durations and length of stay for operating room scheduling, [3] used a stochastic programming approach for nurse scheduling, and [37] applied stochastic programming to appointment scheduling. To the best of our knowledge, stochastic programming has not been applied to multi-appointment planning with uncertain patient routing before. Section 4.1 formulates the problem as a Stochastic Integer Program. In Section 4.2 the recourse model is presented.

### Problem formulation

Before we define the problem, we first introduce some notation, as summarized in Table 1. We use a set notation, where *T* are the time slots, and *S* the clinician types. The first clinician type (*s* = 1) corresponds with the NPs, who have a schedule in which appointments can be scheduled in time slots 1 to |*T*|− 1. The remaining clinician types *s* (*s* ∈ *S*^∗^) are the ones who have schedules in which slots can be pre-booked for regular patients, or are left empty for walk-ins from multi-disciplinary patients in time slots 2 to |*T*|. A clinician type has a capacity *c*_*s*_, which means *c*_*s*_ clinicians of that type are available to see a patient per time slot.

**Table 1:** Notation

Index and set	Definition
$t \in T, t\in \tilde {T}$	time slots that are in regular and overtime respectively, with $\tilde {T} = \{|T|+ 1, |T|+ 2, {\ldots } \}$, and $T^{*} = T \cup \tilde {T} \setminus \{1\}$
*s* ∈ *S*	clinician types, with *S*^∗^ = *S* ∖{1}
*ξ* ∈ Ξ	scenarios
Parameter	Definition
*x* *s* *ξ*	number of multi-disciplinary patients that arrive in scenario *ξ* and are referred to clinician type *s*
*P*_*s*_	proportion of multi-disciplinary patients that will be referred to clinician type *s* for which ${\sum }_{s\in S^{*}}{P_{s}} = 1$ holds
*c*_*s*_	capacity of clinician type *s*
*ϕ* ^*ξ*^	probability of scenario *ξ*, as derived from Eq. 1
*𝜖*_1_,*𝜖*_2_,*𝜖*_3_	objective function weights
Variable	Definition
*Xs*,*tξ*	number of appointment slots reserved for multi-disciplinary patients in scenario *ξ* that will be referred to clinician type *s*
	in time slot *t*
*Y*_*s*, *t*_	number of pre-booked appointment slots scheduled for clinician type *s* in time slot *t*
*O* *s* *ξ*	expected overtime in scenario *ξ* for clinician type *s*
*W* *s* *ξ*	total expected waiting time in scenario *ξ* for multi-disciplinary patients referred to clinician type *s*
*Ls*,*tξ*	queue length in scenario *ξ* at time *t* for clinician type *s*
*I* *s* *ξ*	idle time in scenario *ξ* for clinician type *s*

By the law of large numbers, the number of arrivals that will be referred to clinician type *s* follows a multinomial distribution. Since there is a finite number of possible arrival patterns, we can evaluate the performance of all possible scenarios, relative to their probability masses. For each of these arrival scenarios *ξ*, the probability of occurrence can therefore be calculated using the probability mass function of the multinomial distribution:

1 $$\begin{array}{@{}rcl@{}} \phi^{\xi} &=& P\left( X_{2}^{\xi} = x_{2}^{\xi} \text{, and }...\text{, and }X_{S}^{\xi} = x_{S}^{\xi}\right) \\ &&{\kern7.3pc}= \frac{(T c_{1})!}{{\prod}_{s \in S^{*}}{x_{s}^{\xi}!}} {\prod}_{s \in S^{*}}{P_{s}^{x_{s}^{\xi}}}, \end{array} $$

whereby the sum of all *x*_*i*_ should be equal to the total amount of appointment slots *c*_1_|*T*| of the NP. Note that for |*S*| = 3 this corresponds to the binomial distribution:

2 $$\begin{array}{@{}rcl@{}} \phi^{\xi} = P\left( X_{2}^{\xi} = x_{2}^{\xi} \text{ and } X_{3}^{\xi} = x_{3}^{\xi}\right) = \frac{\left( T c_{1}^{\xi}\right)!}{x_{2}^{\xi}! x_{3}^{\xi}!} P_{2}^{x_{2}^{\xi}} P_{3}^{x_{3}^{\xi}} \\ = \binom{T c_{1}}{x_{2}^{\xi}} P_{2}^{x_{2}^{\xi}} (1-P_{2})^{T c_{1} - x_{2}^{\xi}}. \end{array} $$

To optimize the blueprint schedule for all scenarios, we minimize for all clinicians *s* ∈ *S*^∗^ the expected overtime $O_{s}^{\xi }$, multi-disciplinary patient waiting time $W_{s}^{\xi }$, and the idle time $I_{s}^{\xi }$. To determine the waiting time, we also introduce the queue length $L_{s,t}^{\xi }$ for clinician type *s* in time slot *t*. Note that the queue length in overtime is determined as well, denoted by $L_{s,\tilde {t}}^{\xi }$. The weights for the overtime, waiting time, and idle time objectives are *𝜖*_1_,*𝜖*_2_, and *𝜖*_3_ respectively.

In the stochastic program, all possible referral scenarios are to be evaluated. Therefore, we need two additional decision variables. $X^{\xi }_{s,t}$ is the number of appointments in the agenda of clinician type *s* = 1, that will be referred to clinician type *s* (*s* ∈ *S*^∗^), scheduled in time slot *t* in scenario *ξ*. *Y*_*s*,*t*_ is the number of pre-booked appointments for regular patients for clinician type *s* (*s* ∈ *S*^∗^) in time slot *t*. This variable is independent of the scenarios, since it reflects the tactical level blueprint schedule, which has to be set before the realization of the patient arrivals.

The formal problem definition is as follows:

3 $$ \min \sum\limits_{\xi \in {\Xi}}{\phi^{\xi} \left( \epsilon_{1} \sum\limits_{s \in S^{*}}{O^{\xi}_{s}} + \epsilon_{2} \sum\limits_{s \in S^{*}}{W^{\xi}_{s}} + \epsilon_{3} \sum\limits_{s \in S^{*}}{I^{\xi}_{s}}\right)} $$

s.t.

4 $$\begin{array}{@{}rcl@{}} &&{} \sum\limits_{t \in T}{X^{\xi}_{s,t}} = x^{\xi}_{s} \quad \forall s \in S^{*}, \xi \in {\Xi}, \end{array} $$

5 $$\begin{array}{@{}rcl@{}} &&{} X^{\xi}_{1,t} = 0 \quad \forall t \geq |T|, \end{array} $$

6 $$\begin{array}{@{}rcl@{}} &&{} \sum\limits_{s \in S^{*}}{X^{\xi}_{s,t}} = c_1 \quad \forall t \in T, \xi \in {\Xi}, \end{array} $$

7 $$\begin{array}{@{}rcl@{}} &&{} Y_{s,t} \leq c_s \quad \forall t \in T \setminus \{1\}, s \in S^{*}, \end{array} $$

8 $$\begin{array}{@{}rcl@{}} &&{} Y_{s,1} = c_s \quad \forall s \in S^{*}, \end{array} $$

9 $$\begin{array}{@{}rcl@{}} &&{} L^{\xi}_{s,t} \geq X^{\xi}_{s,t} + Y_{s,t} - c_s \quad \forall s \in S^{*}, \xi \in {\Xi}, t = 1, \end{array} $$

10 $$\begin{array}{@{}rcl@{}} &&{} L^{\xi}_{s,t} \geq L^{\xi}_{s,t-1} + X^{\xi}_{s,t} + Y_{s,t} - c_s \quad \forall t \in T^{*}, s \in S^{*}, \xi \in {\Xi}, \end{array} $$

11 $$\begin{array}{@{}rcl@{}} &&{} O^{\xi}_{s} \geq L^{\xi}_{s,|T|} \quad \forall s \in S^{*}, \xi \in {\Xi}, \end{array} $$

12 $$\begin{array}{@{}rcl@{}} &&{} W^{\xi}_{s} \geq \sum\limits_{t \in T \cup \tilde{T}}{L^{\xi}_{s,t}} \quad \forall s \in S^{*}, \xi \in {\Xi}, \end{array} $$

13 $$\begin{array}{@{}rcl@{}} &&{} I^{\xi}_{s} \geq c_s |T| + O^{\xi}_{s} - \sum\limits_{t \in T}{\left( Y_{s,t} + X^{\xi}_{s,t} \right)} \quad \forall s \in S^{*}, \xi \in {\Xi}, \end{array} $$

14 $$\begin{array}{@{}rcl@{}} &&{} all\; variables \in \mathbb{Z}^{+} . \end{array} $$

The objective is to minimize the weighted overtime, waiting time, and idle time, relative to the probability of each possible scenario of multi-disciplinary patient arrivals (3). For every scenario, the number of appointments to be scheduled for clinician type 1 (e.g. the NP) is given by the population distribution, and thus evaluated for every scenario (4). Note that the final slot of the booking horizon cannot be used by the NP (5). The number of these appointments should be equal to the capacity of this clinician type (6). Also, for the remaining clinician types, the number of pre-booked appointments cannot exceed the capacity (7). Note that the first time slot of the booking horizon, no multi-disciplinary patients can be seen, thus all appointment slots can be filled with pre-booked appointments (8). The queue length equals the queue length of the previous period plus the new arrivals (both walk-in and scheduled) minus the capacity of the clinician type (9)–(10). We assume only one clinician per clinician type to work in overtime, if necessary. Therefore, the number of overtime patients equals the number of overtime slots, which is equal to the queue length of the last time slot for each clinician type (11). Note that this equation can be replaced with (15) to include multiple clinicians serving overtime patients:

15 $$ O^{\xi}_{s} \geq \sum{t \in \tilde{T}}{L^{\xi}_{s,t-1}} \quad \forall s \in S^{*}, \xi \in {\Xi}.  $$

The waiting time for each clinician type is the sum of all queues during the planning horizon, together with the waiting that occurs in overtime (12). Finally, the idle time equals the total time in which the clinicians of a clinician type are unoccupied during the planning horizon (13). Due to the structure of the model, all variables should be nonnegative integers (14).

The number of scenarios |Ξ|, grows with the number of appointment slots *c*|*T*| and the number of clinician types |*S*|, following the multinomial distribution:

16 $$ \left| {\Xi} \right| = \binom{c|T| + (|S|-1) - 1}{(|S|-1) -1}. $$

For a small clinic instance, with 6 time slots with capacity 3, and 5 clinician types, 1,330 scenarios should be evaluated. For instances of clinics with 10 time slots with capacity 4, and 6 clinician types, 123,410 scenarios need to be evaluated. This shows that the problem becomes intractable for large instance sizes, through the high number of scenarios. Therefore, the Sample Average Approximation (SAA) algorithm will be applied, which approximates the objective function by considering a random selection of all possible scenarios [1, 20]. To apply the SAA, we reformulate the stochastic program as a recourse model in Section 4.2.

### Recourse model

In the two-stage stochastic program with recourse, the first stage decides upon the optimal blueprint schedules for the clinician types. This decision is made at the tactical level, and is fixed for every possible scenario. In the second stage, the optimal scheduling strategy for the multi-disciplinary patients at the NP is determined by minimizing the recourse function, given the realization of multi-disciplinary patient arrivals.

Through the recourse formulation, as presented in the ??, it is seen that the recourse model is hard to solve, as it requires a high number of integer recourse functions to be solved. However, the constraint matrix that defines the feasible region of $X_{s,t}^{\xi }$ of the integer recourse function is totally unimodular, as all determinants of the constraint matrix are 0, + 1, or -1, and each column has two non-zero entries, which sum up to 0. Therefore, we can use the LP-relaxation to solve our integer program if the right-hand side is integer. Since all parameters and variables are integer, as *c*_*s*_, |*T*|, and *Y*_*s*,*t*_ are integers, this allows us to use a relaxation of $X_{s,t}^{\xi }$ as a continuous variable between 0 and 1.

## Approximation algorithms

To find a solution to the problem, we first propose to solve the deterministic equivalent of our problem in Section 5.1, by using expected values for all stochastic variables. This is the current practice in our partnering hospital and the literature. However, the stochastic nature of multi-disciplinary patient arrivals is not taken into account in this approach, which leads to solutions that are not robust in practice. Therefore, we apply the Sample Average Approximation (SAA) method in Section 5.2.

### Average scenario

To be able to solve large instances of the stochastic program (3)–(14) from Section 4.1, we evaluate a deterministic version of the model, in which we assume the multi-disciplinary patient arrival rate to follow the (rounded) average scenario. This approach reflects the current hospital practice, where they design the blueprint schedules based on the expected patient flow, rounded to the nearest integer. Furthermore, we think this approach also reflects the approach taken in the literature, where the patient case mix is assumed to be fixed and known.

The objective function of the original problem (3) is replaced by an easier evaluation (17), only considering one single scenario:

17 $$ \min \epsilon_{1} \sum\limits_{s \in S^{*}}{O_{s}} + \epsilon_{2} \sum\limits_{s \in S^{*}}{W_{s}} + \epsilon_{3} \sum\limits_{s \in S^{*}}{I_{s}} $$

This new model will provide a feasible solution to the original problem. Through the elimination of scenario evaluation, the complexity of the model is decreased, and therefore, the model can be evaluated within reasonable time. We assess the expected quality of the solution in reality, by simulating 1,000 realizations of the system, and evaluating the performance of these realizations.

### Sample average approximation algorithm

The SAA algorithm approximates the objective value by evaluating a sample of |*N*| scenarios. The scenarios in the sample are randomly drawn from the scenario population. SAA does not only provide a solution, it also assesses the solution quality. Both lower and upper bounds to the objective of the stochastic program with corresponding optimality gap and confidence intervals are provided [5]. We follow the SAA method as proposed in [1, 5, 20], and refer to them for an in-depth description of this algorithm. The objective value as defined in Eq. 3, can be approximated by the average costs of all selected scenarios (18). This gives the following objective for a given solution $\hat {x}$:

18 $$ \min \frac{1}{|N|} \sum\limits_{n \in N}{\left( \epsilon_{1} \sum\limits_{s \in S^{*}}{{O^{n}_{s}}} + \epsilon_{2} \sum\limits_{s \in S^{*}}{{W^{n}_{s}}} + \epsilon_{3} \sum\limits_{s \in S^{*}}{{I^{n}_{s}}}\right)} $$

The constraints corresponding to the mathematical model of the SAA, are equal to constraints (31)–(39), where the full scenario set Ξ (*ξ* ∈ Ξ) is replaced by a sample set of scenarios *N* (*n* ∈ *N*).

This algorithm generates |*M*| replications of |*N*| samples for which the SAA model is solved. For each replication *m*, we generate a random sample of size |*N*|, and let $\hat {v}_{|N|}^{m}$ be the optimal objective value, and $\hat {x}_{|N|}^{m}$ be the corresponding optimal solution for replication *m*. When these values are computed for all replications, we evaluate statistical bounds over the total number of replications |*M*|. We have:

19 $$ \bar{v}_{|N|}^{|M|} = \frac{1}{|M|} \sum\limits_{m \in M}{\hat{v}_{|N|}^{m}}, $$

which is an estimator of the objective function $E[\hat {v}_{|N|}]$, and thus a lower bound to the optimal solution [20]. Furthermore, we have:

20 $$ Var_{\bar{v}_{|N|}^{|M|}} = \frac{1}{|M|(|M|-1)} \sum\limits_{m \in M}{\left( \hat{v}_{|N|}^{m} - \bar{v}_{|N|}^{|M|}\right)^{2}}, $$

which is an estimator of the variance of $E[\hat {v}_{|N|}]$.

Through the Central Limit Theorem, we can determine the 95 *%* confidence interval (*α* = 0.05) of the lower bound by:

21 $$ \left[ \bar{v}_{|N|}^{|M|} - \frac{z_{a/2}*\sigma_{\bar{v}_{|N|}^{|M|}}}{\sqrt{|N|}}, \quad \bar{v}_{|N|}^{|M|} + \frac{z_{a/2}*\sigma_{\bar{v}_{|N|}^{|M|}}}{\sqrt{|N|}} \right]. $$

Furthermore, an independent random sample of size |*N*^′^| is generated. To compute the upper bound, the independent sample of size |*N*^′^| is used to estimate the true objective value $\hat {g}_{|N^{\prime }|}\left (\hat {x}_{|N|}^{m}\right )$, using (22), and the solution variance ${Var}_{\hat {g}_{|N^{\prime }|}\left (\hat {x}_{|N|}^{m}\right )}$, using (23).

22 $$\begin{array}{@{}rcl@{}} &&\hat{g}_{|N^{\prime}|}(\bar x) \\ &&= \frac{1}{|N^{\prime}|} \sum\limits_{n \in N^{\prime}}{\left( \epsilon_{1} \sum\limits_{s \in S^{*}}{{O^{n}_{s}}} + \epsilon_{2} \sum\limits_{s \in S^{*}}{{W^{n}_{s}}} + \epsilon_{3} \sum\limits_{s \in S^{*}}{{I^{n}_{s}}}\right)} \end{array} $$

23 $$\begin{array}{@{}rcl@{}} &&Var_{\hat{g}_{|N^{\prime}|}(\bar{x})} = \frac{1}{|N^{\prime}| (|N^{\prime}|-1)} \\ &&\sum\limits_{n \in N^{\prime}}{\left[{\left( \epsilon_{1} \sum\limits_{s \in S^{*}}{{O^{n}_{s}}} + \epsilon_{2} \sum\limits_{s \in S^{*}}{{W^{n}_{s}}} + \epsilon_{3} \sum\limits_{s \in S^{*}}{{I^{n}_{s}}}\right)} - \hat{g}_{|N^{\prime}|}(\bar{x}) \right]^{2}} \end{array} $$

We can determine the 95 *%* confidence interval (*α* = 0.05) of the upper bound by:

24 $$ \left[ \hat{g}_{|N^{\prime}|}(\bar x) - \frac{z_{\alpha/2}*\sigma_{\hat{g}_{|N^{\prime}|}(\bar x)}}{\sqrt{|N^{\prime}|}}, \quad \hat{g}_{|N^{\prime}|}(\bar x) + \frac{z_{\alpha/2}*\sigma_{\hat{g}_{|N^{\prime}|}(\bar x)}}{\sqrt{|N^{\prime}|}} \right]. $$

The optimality gap of each feasible solution $\hat {x}_{|N|}^{m}$ can now be estimated by subtracting the lower bound from the upper bound, $\hat {g}_{|N^{\prime }|}\left (\hat {x}_{|N|}^{m}\right ) - \bar {v}_{|N|}^{|M|}$, with corresponding estimated variance $Var_{\bar {v}_{|N|}^{|M|}} + Var_{\hat {g}_{|N^{\prime }|}\left (\hat {x}_{|N|}^{m}\right )}$. Furthermore, a final solution to the problem can be chosen from the replication sample, for example with the best value for $\hat {g}_{|N^{\prime }|}\left (\hat {x}_{|N|}^{m}\right )$.

## Numerical experiments

This section describes the numerical experiments. Section 6.1 describes the test instances and input parameters, and Section 6.2 presents the experiment results.

### Input parameters

This section describes the input parameters and test instances, as summarized in Table 2.

**Table 2:** Experiment settings

Parameter	Settings
|*N*|	25
|*M*|	20
|*N*^′^|	1,000
|*S*|	5
|*T*|	10
*c*	1,2,4
(*P*_2_,…,*P*_5_)	(0.25, 0.25, 0.25, 0.25),
	(0.1, 0.2, 0.3, 0.4), (0.4, 0.1, 0.1, 0.4)
*𝜖*_1_,*𝜖*_2_,*𝜖*_3_	$\frac {1}{3}$

#### Input parameters

We solve the SAA model for sample size |*N*| = 25, number of replications |*M*| = 20, and sample size to estimate the objective value |*N*^′^| = 1,000. The SAA algorithm is implemented in AIMMS 4 with CPLEX 12.6.

#### Test instances

We consider an outpatient clinic with |*S*| = 5 clinician types. Since a clinic operates during the afternoon, in which typically a planning horizon between 8 and 10 time slots of 30 minutes is considered, we use a planning horizon of |*T*| = 10. Hereby we consider a capacity of *c* = {1,2,4}. This way, we vary over *c*|*T*| = 10, 20, or 40 appointment slots per outpatient clinic. 4 treatment specialists are considered, for which we vary over three scenario distributions. These distributions are given by (*P*_2_,…,*P*_5_) = (0.25, 0.25, 0.25, 0.25) for pattern 1, (0.1, 0.2, 0.3, 0.4) for pattern 2, and (0.4, 0.1, 0.1, 0.4) for pattern 3. Recall that multi-disciplinary patients cannot be referred to clinicians of type 1, since these clinicians diagnose the patient. Equal weights are assigned to *𝜖*_1_, *𝜖*_2_, and *𝜖*_3_.

### Experiment results

This section first describes the outcomes of the experiments to set the input parameters. Thereafter, the results of the different test instances are discussed.

#### Input parameter setting experiments

The total number of possible scenarios follows from the number of clinician types and the number of appointment slots, as determined with Eq. 16. Unfortunately, the stochastic program becomes intractable if all possible scenarios are evaluated. Therefore, we determine a reasonable sample size in terms of solution quality and computation time.

**Fig. 3:**
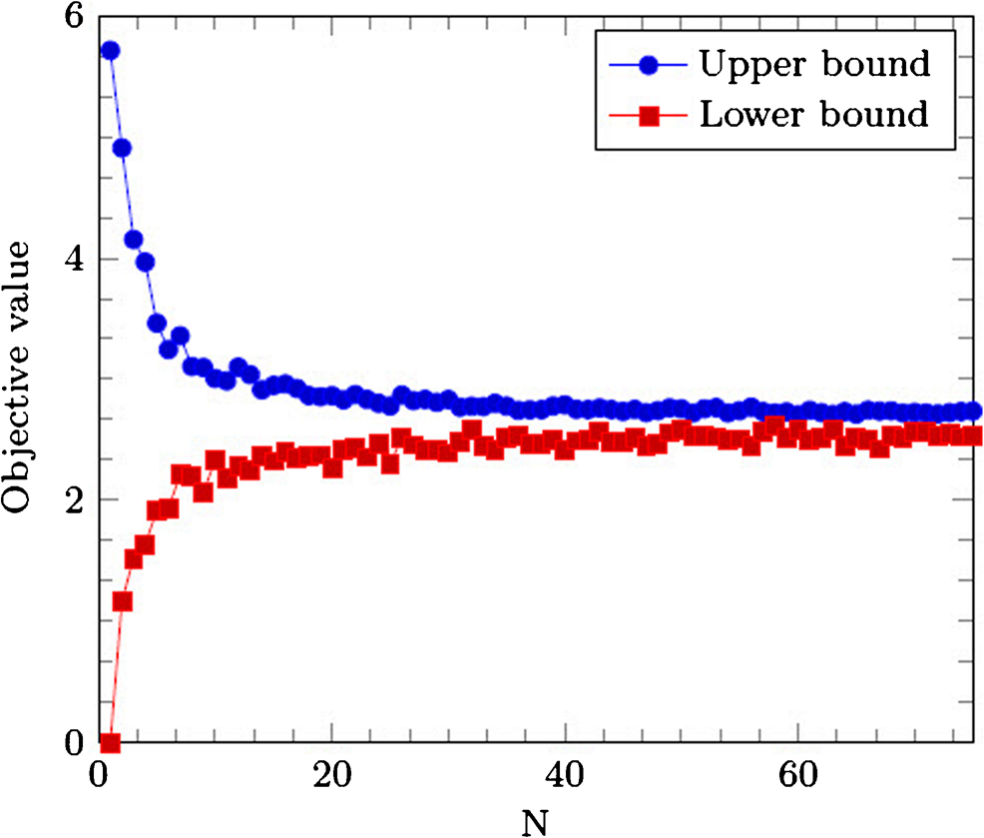
Objective value behavior with increasing number of scenarios |*N*|

To evaluate the amount of samples and replications, we analyze the scenario with Pattern 1 in more detail. Figure 3 shows the objective value behavior with different values for |*N*|. The objective value converges, and it can be seen that |*N*| = 25 samples will provide a reasonable optimality gap.

The solution quality increases with an increased number of samples and increased number of replications, against a price of computation time. For our problem instances, a sample size of |*N*| = 25 and replication number of |*M*| = 20 showed to give good solutions. In the remainder of this research, all experiments are performed with |*N*| = 25 and |*M*| = 20, unless stated otherwise.

#### Experiment results of test instances

Table 3 shows the results of the experiments. We analyzed the difference between the stochastic and deterministic approach, the effect of the clinic size, and the impact of different population distributions.

**Table 3:** Results of numerical experiments

Exp.	Settings				SAA	Det. approach	
no.	*c*	|*T*|	|*cT*|	*p*	Obj.	Obj.	True obj.
1	1	10	10	1	2.200	0	4.616
2	1	10	10	2	1.893	0	5.150
3	1	10	10	3	1.867	0	4.224
4	2	10	20	1	2.613	0	5.651
5	2	10	20	2	2.267	0	6.725
6	2	10	20	3	2.307	0	5.838
7	4	10	40	1	3.293	0	7.217
8	4	10	40	2	3.120	0	9.697
9	4	10	40	3	2.787	0	8.217

**Fig. 4:**
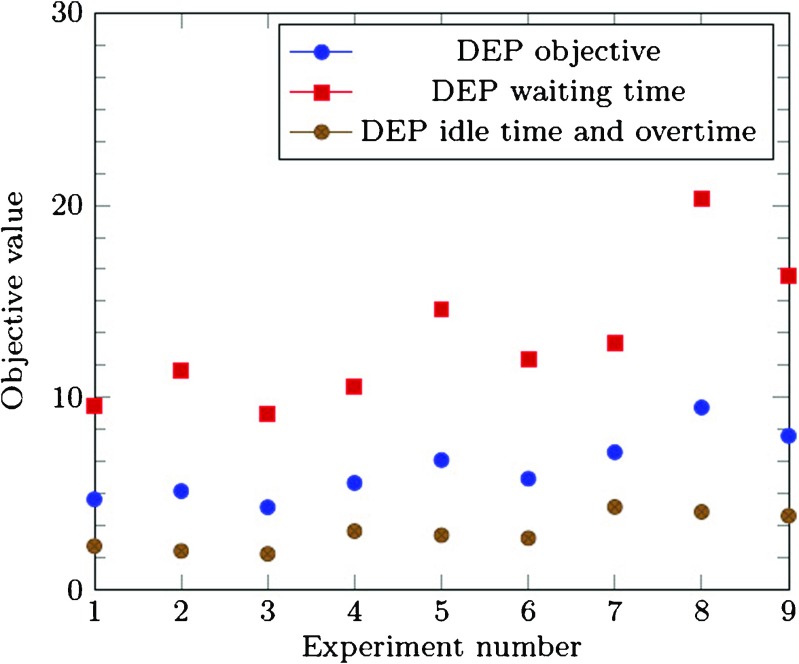
True objective value behavior of the deterministic equivalent problem

As Table 3 shows, the deterministic equivalent problem always derived an objective value of 0. Since only one scenario is evaluated, the schedules of the clinician types can be exactly adapted to the NPs’ schedule. Thus, no waiting time, idle time, and overtime are incurred. However, as we can see in the evaluation of the deterministic equivalent solutions with 1,000 realizations, there will be an equal amount of overtime and idle time, as well as a large amount of waiting time in practice, which adds up to two to three times the performance of the more robust solution of the SAA approach (see Fig. 4). Note that the idle time and overtime are equal, as the deterministic equivalent solution fills all appointment slots. For each incurred idle appointment slot, a patient needs to be seen in overtime. Thus we can conclude that the SAA solution is more robust in practice, as it encounters for uncertainties in arrivals.

When the clinic size increases, the planning performance of the clinic slightly reduces, as can be seen from Table 3. Furthermore, the scenario distribution has impact on the schedule performance. Pattern 1 showed to have worse performance than patterns 2 and 3, which can be explained by the fact that every clinician’s schedule has the same degree of uncertainty. In the other patterns, some clinician types get less referred multi-disciplinary patients, which means less disturbance by multi-disciplinary patients. On the other hand, some clinician types get more referred multi-disciplinary patients, which gives them economies of scale.

## Case study

This section presents a case study of the hepato-pancreato-biliary (HPB) clinic of UMCU. In Section 7.1, UMCU’s HPB clinic is described to give some context. Section 7.2 gives the input parameters and describes the case study instance. Finally, Section 7.3 presents the case study results using the SAA algorithm.

### HPB department

UMCU’s HPB cancer clinic provides care to patients with a (possible) tumor in their liver, pancreas, gallbladder, or biliary. In 2015, 318 new multi-disciplinary patients were seen in this clinic, which faces a growing patient demand. Every Tuesday, an MTM is conducted to assess all multi-disciplinary patients who were referred to UMCU, as well as the patients who need a second-opinion or patients who experienced recurring physical discomfort. Each patient has an intake (and possible additional diagnostic tests) in the morning of the same day. Four different medical specialties are present in the MTM meeting, in line with the possible treatment options: an oncological surgeon, a gastroenterologist, a radiotherapist, and a medical oncologist. Furthermore, the NP, pathologist, radiologist, genetic counselor, and some paramedical staff join the MTM.

During the afternoon, the multi-disciplinary clinic takes place, with consultation possibilities for all four specialties. Since surgery and chemotherapy are the most frequently recommended treatment modes, these specialties are present with multiple staff members. Furthermore, regular patients are seen by the four specialties for follow-up consultations, to ensure a high clinician occupation rate. These patients are pre-scheduled depending on the patient and clinician’s preferences.

UMCU has provided real life data to evaluate the blueprint schedule design for the HPB clinic. The data spans the period of January 2015 to June 2016. Furthermore, the HPB oncology department of UMCU is exploring a growth scenario, in which is collaborated with multiple neighboring hospitals. Therefore, we evaluate this growth scenario as well.

### HPB instances and input parameters

#### Current situation

We consider a small outpatient clinic with |*S*| = 5 clinician types, with a planning horizon of |*T*| = 8, each consisting of 2 time slots of 30 minutes. Thus, the available capacity equals *c* = 2, which gives |*cT*| = 16 appointment slots.

The proportion of a population that requires a specific treatment modality, typically depends on the tumor types and the treatment possibilities per tumor type. Let |*A*| be the number of tumor types, and let the *k*_*a*_ be the proportion of the population with this specific tumor type. The probability that a multi-disciplinary patient with tumor *a* gets treatment *s* is denoted with *p*_*a*,*s*_ (${\sum }_{s\in S^{*}}{p_{a,s}}= 1 \ \forall a \in A$). Therefore, through probability mapping, the proportion *P*_*s*_ of all appointments that will be referred to clinician type *s* can be determined by:

25 $$ P_{s} = \sum\limits_{a \in A}{k_{a} p_{a,s}} \quad \forall s \in S^{*}. $$

We derived the population distribution and referral probabilities from the hospital data. The population distribution is given by (*k*_1_, …, *k*_4_) = (0.21, 0.10, 0.29, 0.40), and Table 4 gives the referral probabilities to the surgeon (surg.), oncologist (onc.), radiotherapist (RT), and gastroenterologist (GE). This gives a scenario distribution of (*P*_2_, …, *P*_5_) = (0.3208, 0.3113, 0.1849, 0.1830).

**Table 4:** Referral probabilities from clinician type 1 to other clinician types per appointment type for the HPB case study

Appt. type	Surg.	Onc.	RT	GE
1	0.46	0.10	0.14	0.30
2	0.38	0.28	0.34	0.00
3	0.38	0.27	0.35	0.00
4	0.19	0.46	0.05	0.30

Since hospital staff was divided on the weights of the three performance measures, we evaluate various weight scenarios, as shown in Table 5.

**Table 5:** Weight settings, including overtime (*𝜖*_1_), waiting time (*𝜖*_2_), and idle time (*𝜖*_3_)

Weight scenario no.	*𝜖*_1_	*𝜖*_2_	*𝜖*_3_
1	$\frac {1}{3}$	$\frac {1}{3}$	$\frac {1}{3}$
2	$\frac {1}{5}$	$\frac {3}{5}$	$\frac {1}{5}$
3	$\frac {2}{5}$	$\frac {1}{5}$	$\frac {2}{5}$
4	$\frac {1}{8}$	$\frac {2}{8}$	$\frac {5}{8}$

To compare the potential savings for UMCU using the results of the model, we also analyze the current way of working, which can be approximated by the deterministic equivalent of the stochastic problem.

#### Growth scenario

As the current outpatient clinic size is rather small, the HPB departments of UMCU and its neighboring hospitals will merge into one multi-disciplinary HPB cancer clinic. In this new clinic, the same number of clinician types and appointment slots are considered (|*S*| = 5 and |*T*| = 8), but each clinician type has capacity *c* = 4. Furthermore, the population will slightly change, to (*k*_1_, …, *k*_4_) = (0.25, 0.11, 0.25, 0.39). We assume the referral probabilities remain the same as in the current situation, which gives (*P*_2_, …, *P*_5_) = (0.3259, 0.3027, 0.1794, 0.1920).

Concluding, nine case study experiments are executed, as shown in Table 6.

**Table 6:** Case study experiment design

Exp.no.	|*S*|	|*T*|	*c*	Population distribution	Weight scenario no.
CS1	5	8	2	(0.3208, 0.3113, 0.1849, 0.1830)	1
CS2	5	8	2	(0.3208, 0.3113, 0.1849, 0.1830)	2
CS3	5	8	2	(0.3208, 0.3113, 0.1849, 0.1830)	3
CS4	5	8	2	(0.3208, 0.3113, 0.1849, 0.1830)	4
DE5	5	8	2	(0.3208, 0.3113, 0.1849, 0.1830)	1
DE6	5	8	2	(0.3208, 0.3113, 0.1849, 0.1830)	2
DE7	5	8	2	(0.3208, 0.3113, 0.1849, 0.1830)	3
DE8	5	8	2	(0.3208, 0.3113, 0.1849, 0.1830)	4
CS9	5	8	4	(0.3259, 0.3027, 0.1794, 0.1920)	1
CS10	5	8	4	(0.3259, 0.3027, 0.1794, 0.1920)	2
CS11	5	8	4	(0.3259, 0.3027, 0.1794, 0.1920)	3
CS12	5	8	4	(0.3259, 0.3027, 0.1794, 0.1920)	4

### Case study results

**Fig. 5:**
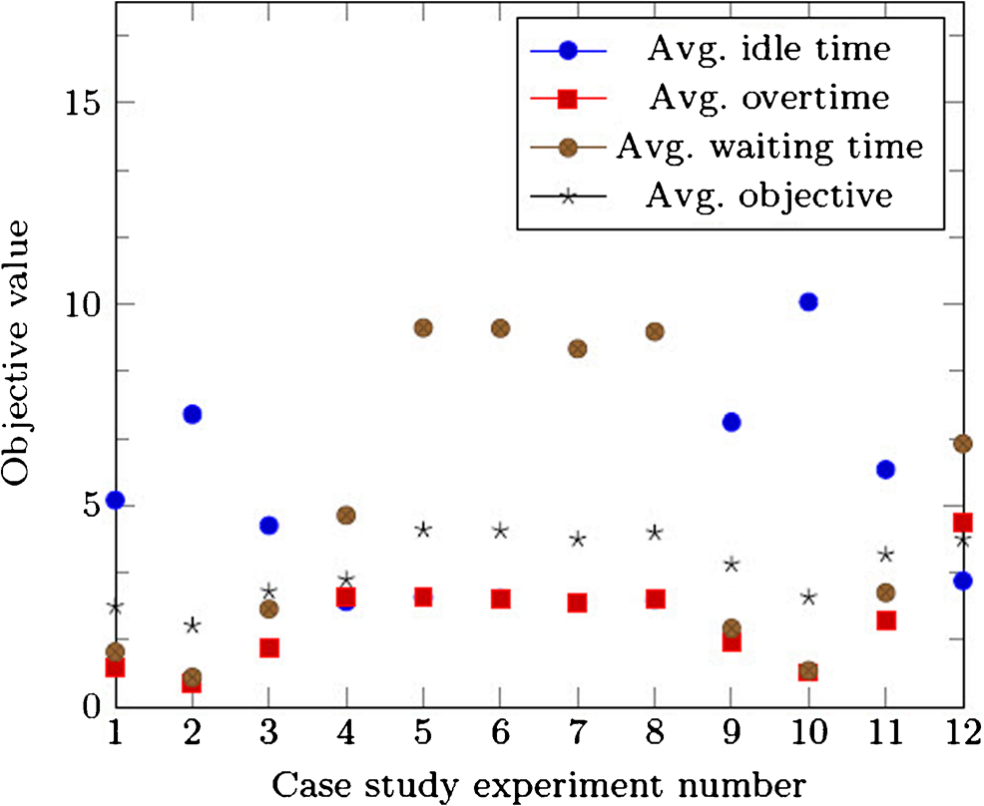
Results of case study experiments

The results of the case study experiments are shown in Fig. 5. Note that for the deterministic equivalent experiments, the true objective value is shown.

In both the current situation and the growth scenario, the SAA approach found good quality solutions for the different weight patterns, as the gap between the upper bound and lower bound is reasonably small. Better performance for specific performance indicators can be derived, depending on the weight settings. However, a higher weight on specific indicators comes at a cost of reduced performance on the other indicators. More specifically, a trade-off has to be made between waiting and overtime, and the idle time, as solutions with better waiting and overtime often face worse idle time performance.

In the growth scenario more multi-disciplinary patients are seen by the clinicians, which reduces the uncertainty in their schedules. This is reflected in the lower SAA objective values for the growth scenario compared to the current situation relative to the size of the clinic (a difference of factor 1.48). However, through the higher amount of staff and patients, higher absolute total overtime, idle time, and waiting time are incurred.

Compared with the current way of working, all proposed SAA solutions show better overall performance, of 50 minutes on average, which correspond with associated savings of 21% of the total clinic time. The performance on overtime and waiting time is improved for all weight settings, with up to 260 minutes less waiting time in total. However, in terms of idle time, the current situation might outperform the proposed SAA solutions. This is caused by the different amount of regular patients that are pre-booked in the the multi-disciplinary clinic. As can be seen from the figure, more patients are served in the current situation, as the overtime minus the idle time is greater than the overtime minus the idle time in the SAA solutions. However, the UMCU decision makers do not aim to serve as many regular patients as possible, but to serve all of their patients with as few waiting time and overtime as possible, given a reasonable idle time performance. The idle time performance is influenced by the amount of regular patients scheduled, the more regular patients, the less idle time. If idle time is not important at all, no regular patients will be scheduled, as this way the waiting and overtime are minimized. Therefore, the weight given to the idle time, includes the weight given for serving a high amount of patients. Note that the SAA solution for experiment 4 shows improved performance on all performance indicators, including the idle time, through the high weight on idle time. This shows that the SAA approach is capable of finding better overall schedules than the current way of working.

Concluding, we were able to find good schedules for the HPB clinic practice, based on various weight settings. The clinic has to decide which weight setting is important to them, as the overtime, idle time and waiting time measures vary according to specific settings. Furthermore, they have to make a final decision on which blueprint schedule to implement.

## Conclusion and discussion

This paper considers a two-stage stochastic program with integer recourse for the scheduling of multi-disciplinary cancer clinics. To solve this scheduling problem, an SAA approach is adopted. Numerical experiments show and that the amount of uncertainty in patient arrivals influences the possible performance of a clinic, and that both for theory and practice good schedules can be obtained using this approach, which improves the current situation with 21% on average.

Health care practitioners should carefully discuss how to set the weights for waiting, idle and overtime, as these affect the resulting schedules. In situations where clinics do not incorporate uncertainty in patient routing, and determine their schedule on the average patient mix, such as in UMCU’s current situation, high weight is (unintentionally) put on idle time, as a high utilization is striven for. However, this might not reflect a clinic’s intentions, which shows a thorough analysis of the current clinic’s schedules is required.

Since all multi-disciplinary patients are discussed at the MTM, this research considers offline planning. In UMCU, the required treatment for all multi-disciplinary patients is known before the scheduling of multi-disciplinary patients in the NPs’ agendas takes place, as this scheduling step is done during the briefing preceding the clinic. In UMCU’s practice, this situation therefore reflects reality. However, in a more general situation, one might want to schedule each multi-disciplinary patient at the time of their appointment request. This requires online planning, for which the stochastic model still can be used. The totally unimodular property needs to be dropped in this case, as the required treatment is not known at the time of the appointment request. Further research should be executed to develop a multiple-stage stochastic program in which this new stochastic variable is taken into account as well.

This research was based on a few assumptions. First, we assumed a fixed slot structure for the blueprint of all clinicians. However, it is known that blueprint schedules without predefined slot structures might result in better performance [8]. Further research should show which slot structure is preferred for multi-disciplinary clinics.

Second, we considered a fixed clinic capacity, an unlimited demand of regular patients, and a fixed amount of multi-disciplinary patients. In practice, demand for multi-disciplinary care varies over the weeks. Hospitals tend to handle this varying demand in several ways. We chose to fix the capacity, and postpone multi-disciplinary patients that arrive after all slots are filled to next week’s clinic. Another way is to always accept multi-disciplinary patients that arrive, and serve them in overtime. In this case, one could adapt the number of appointment slots at the NP in such a way, that it covers the maximum demand in for example 95% of the cases, and add an extra constraint to minimize the number of overbooked NP slots if possible.

Third, we assumed a fixed service duration for all patients. Although service duration variability has an impact on the performance of health care clinics [see e.g. 30], in our case study data on the amount of service duration variability was not known. Furthermore, as our model would explode when adding all sources of variability, we chose to incorporate uncertainty in patient routing over uncertainty in service duration, as the impact of a patient not visiting a provider is higher than the impact of a patient having a shorter visit with a provider. Further research is required to incorporate more sources of variability into one model, such as variability in patient arrivals, service durations, patient routing, and capacity availability.

Fourth, the objective function of our model includes multi-disciplinary patient waiting time, and clinician idle time and overtime. We chose to not take patient access time into account, for multi-disciplinary as well as regular patients. Since all multi-disciplinary patients are assumed to already be present in the hospital, all multi-disciplinary patients have equal arrival times. Including the access time for multi-disciplinary patients would therefore not influence the optimal solution. Furthermore, the access time of regular patients is influenced by factors outside the system under review, as regular patients are also served in other clinics. Therefore, the access time for regular patients cannot be accurately determined.

Fifth, the model assumes that referrals can only be done to clinicians of other types. In health care settings, it might be the case that the clinician who gives the diagnosis, is also one of the treating clinicians. For example the surgeon or the gastroenterologist can have this double function in both the diagnostic as well as the treatment phase. Further research should be done to analyze the effect of recurrent referrals.

Sixth, we assumed patients are served on a FCFS basis. However, in a clinic environment it is debatable whether FCFS is the most equitable priority rule for patients, as patients have diverse priorities, due dates, and appointment series. Furthermore, it is questionable whether it is necessary to use FCFS, as long as patients are served within a reasonable time. As we analyzed a multi-disciplinary clinic with patients with two sequential appointments, the FCFS priority rule is feasible. In a multi-disciplinary clinic with varying numbers of appointments (e.g. patients that can have 2, 3 or 4 appointments in a row), other priority rules, for example based on the expected remaining throughput time, might be more suitable.

Seventh, we analyzed the multi-disciplinary clinic independent from the morning processes. Incorporating the effect of appointments in the morning into the afternoon schedule, or jointly optimizing the morning and afternoon clinics might give improved results which necessitates multi-appointment scheduling solutions with three or more appointments.

Based on our research, several directions for implementation in practice are present. First, the blueprint schedule solution can be implemented, which shows schedulers in which appointment slots a regular patient can be scheduled, and which appointment slots should be left empty, to encounter for multi-disciplinary patients. Second, the second stage model can be used to plan the multi-disciplinary patients in the agenda of the NPs, after the MTM. Currently, UMCU implemented a new blueprint schedule and use simple planning rules for real-time scheduling based on the results of this research.

Since the patient population of a hospital changes over time, and since new treatment modalities can be introduced, the model should be used by hospitals in a dynamic way. We advise hospital managers to redesign their blueprint schedules at least once a year. Our integrated optimization approach, in which all appointment schedules are jointly optimized, can help hospital managers to efficiently organize their multi-disciplinary care systems.

## Appendix: Recourse model

The stochastic problem of Eqs. 3–14 can be formulated as the following recourse model:

26 $$ \text{min } E[Q(x,\xi)], $$

s.t.

27 $$\begin{array}{@{}rcl@{}} && Y_{s,t} \leq c_s \quad \forall t \in T \setminus \{1\}, s \in S^{*}, \end{array} $$

28 $$\begin{array}{@{}rcl@{}} && Y_{s,1} = c_s \quad \forall s \in S^{*}, \end{array} $$

29 $$\begin{array}{@{}rcl@{}} && Y_{s,t} \in \mathbb{Z}^{+} \quad \forall t \in T, s \in S^{*}, \end{array} $$

where *E*[*Q*(*x*,*ξ*)] is the corresponding recourse function, with:

30 $$ Q(x,\xi) = \min \epsilon_{1} \sum\limits_{s \in S^{*}}{O^{\xi}_{s}} + \epsilon_{2} \sum\limits_{s \in S^{*}}{W^{\xi}_{s}} + \epsilon_{3} \sum\limits_{s \in S^{*}}{I^{\xi}_{s}}, $$

s.t.

31 $$\begin{array}{@{}rcl@{}} && \sum\limits_{t \in T}{X^{\xi}_{s,t}} = x^{\xi}_{s} \quad \forall s \in S^{*}, \end{array} $$

32 $$\begin{array}{@{}rcl@{}} && X^{\xi}_{1,t} = 0 \quad \forall t \geq |T|, \end{array} $$

33 $$\begin{array}{@{}rcl@{}} && \sum\limits_{s \in S^{*}}{X^{\xi}_{s,t}} = c_1 \quad \forall t \in T, \end{array} $$

34 $$\begin{array}{@{}rcl@{}} && L^{\xi}_{s,t} \geq X^{\xi}_{s,t} + Y_{s,t} - c_s \quad \forall s \in S^{*}, t = 1, \end{array} $$

35 $$\begin{array}{@{}rcl@{}} && L^{\xi}_{s,t} \geq L^{\xi}_{s,t-1} + X^{\xi}_{s,t} + Y_{s,t} - c_s \quad \forall t \in T^{*}, s \in S^{*}, \end{array} $$

36 $$\begin{array}{@{}rcl@{}} && O^{\xi}_{s} \geq L^{\xi}_{s,|T|} \quad \forall s \in S^{*}, \end{array} $$

37 $$\begin{array}{@{}rcl@{}} && W^{\xi}_{s} \geq \sum\limits_{t \in T}{L^{\xi}_{s,t}} + \sum\limits_{\tilde{t} \in \tilde{T}}{L^{\xi}_{s,\tilde{t}}} \quad \forall s \in S^{*}, \end{array} $$

38 $$\begin{array}{@{}rcl@{}} && I^{\xi}_{s} \geq c_s |T| + O^{\xi}_{s} - \sum\limits_{t \in T}{\left( Y_{s,t} + X^{\xi}_{s,t} \right)} \quad \forall s \in S^{*}, \end{array} $$

39 $$\begin{array}{@{}rcl@{}} && all\; variables \in \mathbb{Z}^{+} . \end{array} $$

## References

[1] Ahmed S, Shapiro A (2002). The sample average approximation method for stochastic programs with integer recourse. SIAM J Optim.

[2] Arnaout A, Smylie J, Seely J, Robertson S, Knight K, Shin S, Ramsey T, Mallick R, Watters J (2013). Improving breast diagnostic services with a rapid access diagnostic and support (rads) program. Ann Surg Oncol.

[3] Bagheri M, Devin A, Izanloo A (2016). An application of stochastic programming method for nurse scheduling problem in real word hospital. Comput Indus Eng.

[4] Balasubramanian H, Biehl S, Dai L, Muriel A (2014). Dynamic allocation of same-day requests in multi-physician primary care practices in the presence of prescheduled appointments. Health Care Manag Sci.

[5] Bentaha M, Battaïa O, Dolgui A (2014). A sample average approximation method for disassembly line balancing problem under uncertainty. Comput Oper Res.

[6] Braaksma A (2015) Timely and efficient planning of treatments through intelligent scheduling. University of Twente, Enschede

[7] Cayirli T, Veral E (2003). Outpatient scheduling in health care: a review of literature. Prod Oper Manag.

[8] Chakraborty S, Muthuraman K, Lawley M (2013). Sequential clinical scheduling with patient no-show: the impact of pre-defined slot structures. Socioecon Plann Sci.

[9] Dharmadhikari N, Zhang J (2013). Simulation optimization of blocking appointment scheduling policies for multi-clinic appointments in centralized scheduling systems. Int J Eng Innov Technol.

[10] Dobson G, Hasija S, Pinker E (2011). Reserving capacity for urgent patients in primary care. Prod Oper Manag.

[11] El-Sharo M, Zheng B, Yoon S, Khasawneh M (2015). An overbooking scheduling model for outpatient appointments in a multi-provider clinic. Oper Res Health Care.

[12] Garey M, Johnson D, Sethi R (1976). The complexity of flowshop and jobshop scheduling. Math Oper Res.

[13] van der Geer S, Frunt M, Romero H, Dellaert N, Jansen-Vullers M, Demeyere T, Neumann M, Krekels G (2012). One-stop-shop treatment for basal cell carcinoma, part of a new disease management strategy. J Eur Acad Dermatol Venereol.

[14] Geerlings R, Aldenkamp A, Gottmer-Welschen L, de With P, Zinger S, van Staa A, de Louw A (2016). Evaluation of a multidisciplinary epilepsy transition clinic for adolescents. Eur J Paediatric Neurol.

[15] Goodridge A, Woodhouse D, Barbour J (2013). Improving patient access at a movement disorder clinic by participating in a process improvement program. BMJ Qual Improv Rep.

[16] Gupta D, Denton B (2008). Appointment scheduling in health care: challenges and opportunities. IIE Trans.

[17] Hulshof P, Kortbeek N, Boucherie R, Hans E, Bakker P (2012). Taxonomic classification of planning decisions in health care: a structured review of the state of the art in or/ms. Health Syst.

[18] Kapamara T, Sheibani K, Petrovic D, Haas O, Reeves C (2007) A simulation of a radiotherapy treatment system: A case study of a local cancer centre. In Proceedings of the ORP3 conference. Minho University, pp 29–35

[19] Klassen K, Rohleder T (1996). Scheduling outpatient appointments in a dynamic environment. J Oper Manag.

[20] Kleywegt A, Shapiro A, Homem-de Mello T (2002). The sample average approximation method for stochastic discrete optimization. SIAM J Optim.

[21] Kortbeek N, Zonderland M, Braaksma A, Vliegen I, Boucherie R, Litvak N, Hans E (2014). Designing cyclic appointment schedules for outpatient clinics with scheduled and unscheduled patient arrivals. Perform Eval.

[22] Leeftink A, Boucherie R, Hans E, Verdaasdonk M, Vliegen I, van Diest P (2016) Batch scheduling in the histopathology laboratory. Flex Serv Manuf J. 10.1007/s10696-016-9257-3 10.1136/jclinpath-2015-203349 26797408

[23] Liang B, Turkcan A, Ceyhan M, Stuart K (2015). Improvement of chemotherapy patient flow and scheduling in an outpatient oncology clinic. Int J Prod Res.

[24] Litton G, Kane D, Clay G, Kruger P, Belnap T, Parkinson B (2010). Multidisciplinary cancer care with a patient and physician satisfaction focus. J Oncol Pract.

[25] Ma X, Sauré A, Puterman ML, Taylor M, Tyldesley S (2016). Capacity planning and appointment scheduling for new patient oncology consults. Health Care Manag Sci.

[26] Matta M, Patterson S (2007). Evaluating multiple performance measures across several dimensions at a multi-facility outpatient center. Health Care Manag Sci.

[27] Min D, Yih Y (2010). Scheduling elective surgery under uncertainty and downstream capacity constraints. Eur J Oper Res.

[28] Murray M, Tantau C (1999). Redefining open access to primary care. Manag Care Q.

[29] Mutlu S, Benneyan J, Terrell J, Jordan V, Turkcan A (2015). A co-availability scheduling model for coordinating multi-disciplinary care teams. Int J Prod Res.

[30] Oh H, Muriel A, Balasubramanian H, Atkinson K, Ptaszkiewicz T (2013). Guidelines for scheduling in primary care under different patient types and stochastic nurse and provider service times. IIE Trans Healthcare Syst Eng.

[31] Oh H, Muriel A, Balasubramanian H (2014) A user-friendly excel simulation for scheduling in primary care practices. In: 2014 Winter Simulation conference (WSC). IEEE, pp 1177–1185

[32] Peng Y, Qu X, Shi J (2014). A hybrid simulation and genetic algorithm approach to determine the optimal scheduling templates for open access clinics admitting walk-in patients. Comput Indus Eng.

[33] Petrovic D, Morshed M, Petrovic S (2011). Multi-objective genetic algorithms for scheduling of radiotherapy treatments for categorised cancer patients. Expert Syst Appl.

[34] Pinedo M, Zacharias C, Zhu N (2015). Scheduling in the service industries: an overview. J Syst Sci Syst Eng.

[35] Qu X, Rardin R, Williams J, Willis D (2007). Matching daily healthcare provider capacity to demand in advanced access scheduling systems. Eur J Oper Res.

[36] Qu X, Rardin R, Williams J (2012). A mean–variance model to optimize the fixed versus open appointment percentages in open access scheduling systems. Decis Support Syst.

[37] Qu X, Peng Y, Kong N, Shi J (2013). A two-phase approach to scheduling multi-category outpatient appointments. a case study of a women’s clinic. Health Care Manag Sci.

[38] Qu X, Peng Y, Shi J, LaGanga L (2015). An mdp model for walk-in patient admission management in primary care clinics. Int J Prod Econ.

[39] Robinson L, Chen R (2010). A comparison of traditional and open-access policies for appointment scheduling. Manuf Serv Oper Manag.

[40] Romero H, Dellaert N, van der Geer S, Frunt M, Jansen-Vullers M, Krekels G (2013). Admission and capacity planning for the implementation of one-stop-shop in skin cancer treatment using simulation-based optimization. Health Care Manag Sci.

[41] Rossi A, Puppato A, Lanzetta M (2013). Heuristics for scheduling a two-stage hybrid flow shop with parallel batching machines: application at a hospital sterilisation plant. Int J Prod Res.

[42] Sadki A, Xie X, Chauvin F (2011) Appointment scheduling of oncology outpatients. In: 2011 IEEE conference on automation science and engineering (CASE). IEEE, pp 513–518

[43] Santibáñez P, Chow V, French J, Puterman M, Tyldesley S (2009). Reducing patient wait times and improving resource utilization at british columbia cancer agency’s ambulatory care unit through simulation. Health Care Manag Sci.

[44] Saremi A, Jula P, ElMekkawy T, Wang G (2015). Bi-criteria appointment scheduling of patients with heterogeneous service sequences. Expert Syst Appl.

[45] Truong V (2015). Optimal advance scheduling. Manag Sci.

[46] Tsai P, Teng G (2014). A stochastic appointment scheduling system on multiple resources with dynamic call-in sequence and patient no-shows for an outpatient clinic. Eur J Oper Res.

[47] Veenhuizen R, Tibben A (2009). Coordinated multidisciplinary care for huntington’s disease. an outpatient department. Brain Res Bull.

[48] Veenhuizen R, Kootstra B, Vink W, Posthumus J, van Bekkum P, Zijlstra M, Dokter J (2011). Coordinated multidisciplinary care for ambulatory huntington’s disease patients. evaluation of 18 months of implementation. Orphanet J Rare Dis.

[49] Wiesche L, Schacht M, Werners B (2017). Strategies for interday appointment scheduling in primary care. Health Care Manag Sci.

